# Mucinous carcinoma occurring in the male breast

**DOI:** 10.3892/ol.2013.1730

**Published:** 2013-12-05

**Authors:** MITSUAKI ISHIDA, TOMOKO UMEDA, YUKI KAWAI, TSUYOSHI MORI, YOSHIHIRO KUBOTA, HAJIME ABE, MUNEO IWAI, KEIKO YOSHIDA, AKIKO KAGOTANI, TOHRU TANI, HIDETOSHI OKABE

**Affiliations:** 1 Department of Clinical Laboratory Medicine and Division of Diagnostic Pathology, Shiga University of Medical Science, Otsu, Shiga 520-2192, Japan; 2 Department of Surgery, Shiga University of Medical Science, Otsu, Shiga 520-2192, Japan

**Keywords:** mucinous carcinoma, male breast, estrogen receptor

## Abstract

Male breast carcinoma is an uncommon neoplasm, accounting for 0.6% of all breast carcinomas. Invasive ductal carcinoma of no special type is the most common type of male breast carcinoma, and mucinous carcinoma occurring in the male breast is extremely rare. In the present study, we report a case of mucinous carcinoma of the male breast and discuss the clinicopathological features of this type of tumor. A 63-year-old Japanese male presented with a gradually enlarged nodule in the right breast. The resected breast specimen revealed pure mucinous carcinoma and immunohistochemical analyses demonstrated that tumor cells were positive for estrogen receptor (ER), but negative for progesterone receptor (PgR). In addition, HER2 expression was not amplified. Pure mucinous carcinoma is generally associated with a low incidence of lymph node or distant metastases, and excellent disease-free survival in females. However, certain cases of this type of tumor with axillary lymph node metastasis in the male breast have been reported. In addition, the immunoprofiles of mucinous carcinoma in males are fundamentally the same as those in females. More than 90% of cases show positive immunoreactivity for ER and/or PgR, and HER2 expression is not amplified. However, it has been reported that breast cancer in males is more frequently positive for ER than in females, and has less HER2 overexpression. The high rate of hormone receptor-positive breast cancer in males is considered to be due to similar conditions as those in breast cancer in postmenopausal women. The pathogenesis of male breast carcinoma, including mucinous carcinoma, remains unclear; therefore, additional clinicopathological studies are required.

## Introduction

Male breast cancer is an uncommon neoplasm, accounting for 0.6% of all breast carcinomas and <1% of malignancies in men (1,2). Carcinoma *in situ* and invasive carcinoma may occur in the male breast. Ductal carcinoma *in situ* is reported in ≤10% of breast carcinomas in males (1). Invasive ductal carcinoma of no special type is the most common type of male breast carcinoma, accounting for ~90%. Mucinous carcinoma, also referred to as colloid carcinoma or gelatinous carcinoma, is histopathologically characterized by the presence of clusters of neoplastic cells suspended in extensive extracellular mucin, and accounts for ~2% of female breast carcinomas (3). However, its occurrence in the male breast is extremely rare (2,4,5). Mucinous carcinoma is histopathologically subclassified into pure and mixed types. The pure form is defined as a lesion with a mucinous carcinoma component of >90% of the tumor; the mixed type is defined as having mucinous and conventional invasive ductal carcinoma components (3). It is well-recognized that pure mucinous carcinoma is generally associated with low rates of recurrence and excellent survival rates. In the present study, we report a case of pure mucinous carcinoma occurring in a male breast and review the clinicopathological features of this extremely rare tumor. Furthermore, we also discuss the subclassification of mucinous carcinoma and the immunohistochemical differences between male and female breast cancer.

## Case report

### Case presentation

A 63-year-old Japanese male without a history of carcinoma, including in the gastrointestinal tract, presented to Shiga University of Medical Science (Otsu, Japan) with a gradually enlarged nodule in the right breast. Physical examination revealed a relatively well-circumscribed nodule, measuring 15×10 mm in diameter, in the right breast. Total resection of the breast nodule with right sentinel lymph node dissection was performed. The patient provided written informed consent.

### Immunohistochemistry

The formalin-fixed, paraffin-embedded tissue blocks of the resected breast specimen and lymph nodes were cut into 3-μm-thick sections, deparaffinized and rehydrated. Each section was stained with hematoxylin and eosin, and then used for immunostaining. Immunohistochemical analyses were performed using an autostainer (XT system Benchmark; Ventana Medical System, Tucson, AZ, USA) according to the manufacturer's instructions. The following primary antibodies were used: mouse monoclonal antibody against estrogen receptor (ER; clone 6F11; Novocastra Laboratories, Ltd., Newcastle upon Tyne, UK) and mouse monoclonal antibody against progesterone receptor (PgR; clone PgR636; DakoCytomation; Dako, Glostrup, Denmark). In addition, immunohistochemical analysis for c-erbB-2 (HER2) oncoprotein was performed using Dako HercepTest II (Dako).

### Histopathological results

**Figure 1:**
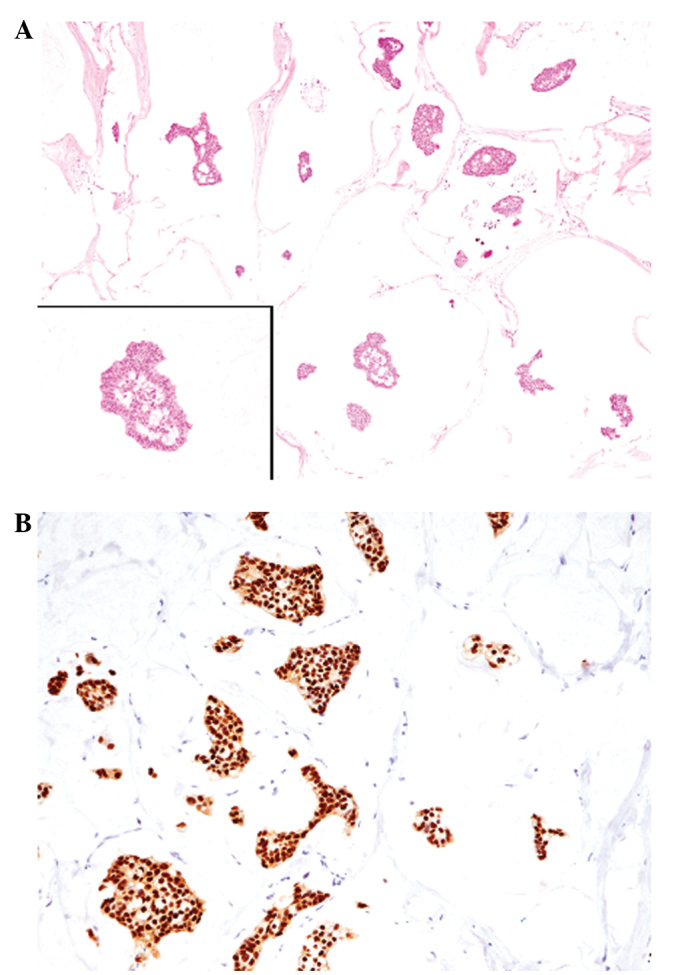
Histopathological and immunohistochemical features of the breast nodule. (A) Clusters of uniform neoplastic cells are floating within rich mucinous material. The neoplastic cells have enlarged round nuclei (inset). Hematoxylin and eosin staining; magnification, ×40 (inset, ×100). (B) Immunostain showing that the estrogen receptor is diffusely expressed (magnification, ×200).

Histopathological study of the resected breast tissue revealed a relatively well-circumscribed nodular lesion in the breast. Clusters of uniform neoplastic cells with slightly enlarged round nuclei containing small nucleoli were suspended within rich mucinous material (mucous lake; Fig. 1A). The tumor had invaded into the surrounding fatty tissue, however, no skin involvement was observed. Neither conventional invasive carcinoma nor intraductal components were present.

### Immunohistochemical results

Immunohistochemical analysis showed that the tumor cells were diffusely positive for ER (Fig. 1B), but negative for PgR. The HER2 score was 0. The sentinel lymph nodes had no metastatic carcinoma. Therefore, an ultimate diagnosis of pure mucinous carcinoma occurring in the male breast was made.

## Discussion

Mucinous carcinoma is histopathologically subclassified into pure and mixed types (3). The pure form is defined as a lesion with a mucinous carcinoma component in more than 90% of the tumor, and the mixed type has both mucinous and conventional invasive carcinoma components (3). In the present case, a diagnosis of pure mucinous carcinoma was made since no conventional invasive ductal carcinoma component was present. It has been reported that the prognosis of pure mucinous carcinoma is more favorable than that of mixed type in females. Pure mucinous carcinoma in females is associated with a low incidence of nodal metastasis (2–4%) (6,7), and the 10-year overall survival ranges from 80 to 100% (3). Therefore, certain researchers have suggested that axillary lymph node dissection may be unnecessary for pure mucinous carcinoma, and they recommend sentinel lymph node dissection instead for patients with this form of tumor (6). However, certain cases of pure mucinous carcinoma with axillary lymph node metastasis in the male breast have been reported (5,8,9), and a case of pure mucinous carcinoma with lung metastasis in the male breast has also been documented (10). Thus, sentinel lymph node technique and clinical follow-up are considered necessary for patients with mucinous carcinoma.

Cytological examination by fine needle aspiration (FNA) is an easy and useful procedure for the diagnosis of breast tumors. The cytological features of mucinous carcinoma include the presence of relatively uniform neoplastic cells with slightly enlarged round to oval nuclei containing small nucleoli arranged in cords or small nests, within a rich mucinous material (11). A few cases of mucinous carcinoma of the male breast successfully diagnosed by FNA have been reported (8,12–14), although FNA was not performed in the present case. Recently, Ingle *et al* documented a case of pure mucinous carcinoma with axillary lymph node metastasis in a 75-year-old male (8). The authors reported that the breast tumor and lymph node metastasis were successfully diagnosed as mucinous carcinoma by preoperative FNA (8). These results suggest that FNA is also a useful tool for detecting male mucinous carcinoma, even in cases with lymph node metastasis.

Capella *et al* classified mucinous carcinoma based on structural and cytological features as type A (paucicellular; a tumor showing a ribbon, annular or cribriform growth pattern with prominent extracellular mucin) and type B (hypercellular; a tumor showing clump- or sheet-like structures with reduced extracellular mucin) (15). According to this classification, the present case falls into the category of type A. It is well-known that type B mucinous carcinoma frequently shows neuroendocrine differentiation (15,16). Until now, only a few cases of neuroendocrine carcinoma with a mucinous carcinoma component within the same tumor have been reported in the female breast (17,18), although this type of tumor has not been documented yet in the male breast. This phenomenon is suggested to represent the same genetic background present in both type B mucinous carcinoma and neuroendocrine carcinoma of the breast, since Weigelt *et al* clearly revealed that no differences in gene expression were present in these two types of tumors using genome-wide oligonucleotide microarrays (19).

The immunoprofiles of mucinous carcinoma in males are fundamentally the same as those in females. More than 90% of cases show positive immunoreactivity for ER and/or PgR, and HER2 expression is not amplified, as observed in the present case (5,8,9). However, Muir *et al* reported that breast cancer in males is more frequently positive for ER than in females (male 81% vs. female 69%) and has lower HER2 overexpression (5% vs. 17%, respectively), but no significant difference in PgR (63% vs. 56%, respectively) (20). Postmenopausal women have been identified to present with breast cancer that is more likely to have hormone receptor expression. One possibility is that hormone receptor-positive breast cancer is a consequence of aberrant steroid receptor upregulation in the estrogen-starved postmenopausal setting (20). Therefore, the high rate of hormone receptor-positive breast cancer in males is also likely due to similar conditions as breast cancer in postmenopausal women. The pathogenesis of male breast carcinoma, including mucinous carcinoma, remains unclear; therefore, additional clinicopathological studies are required.
